# Time-resolved transcriptomic analysis suggests candidate hub gene modules and putative regulatory pathways in tobacco under dicamba stress

**DOI:** 10.3389/fpls.2026.1850240

**Published:** 2026-07-13

**Authors:** Zhaopeng Luo, Lifeng Jin, Peilin Li, Zefeng Li, Chaonan Shi, Mingzhu Wu, Xin Xu, Xiaozong Wu, Zhong Wang, Meng Li, Jun Yang, Chan Qiao, Feng Li

**Affiliations:** 1 China Tobacco Gene Research Center (CTGRC), Zhengzhou Tobacco Research Institute of China National Tobacco Corporation (CNTC), Zhengzhou, China; 2 International Joint Research Laboratory for Utilization of Plant Functional Components, College of Tobacco Science and Engineering, Zhengzhou University of Light Industry, Zhengzhou, China; 3 Institute of Tobacco Science, Heilongjiang Provincial Tobacco Company of China National Tobacco Corporation (CNTC), Harbin, China

**Keywords:** dicamba, *Nicotiana tabacum*, NtIAA protein family, predicted regulatory network, weighted gene co-expression network analysis (WGCNA)

## Abstract

**Introduction:**

Widespread dicamba use poses challenges of resistance and phytotoxicity. To investigate the temporal molecular response mechanisms of tobacco, this study performed a time-resolved transcriptomic analysis of tobacco seedlings exposed to dicamba.

**Methods:**

Samples were collected at 6, 24, and 72 h after treatment.

**Results:**

Results indicated that tobacco exhibits a putative three-phase transcriptional adaptation pattern of “perception-defense-repair”. In the early stage (6h), *NtIAA* genes were rapidly induced alongside activation of the glutathione system, potentially alleviating oxidative stress; at the mid-stage (24h), enhanced carotenoid synthesis and thylakoid reconstruction appeared to protect photosynthetic structures; at the late stage (72h), the transcriptional response shifted toward systemic repair through secondary metabolism, including phenylpropanoid biosynthesis. Predictive regulatory network analysis suggested that the ERF transcription factor *Nitab4.5_0000015g0020* may act as a candidate hub, potentially linking auxin signaling and ribosomal protein genes.

**Discussion:**

Taken together, this study provides transcriptomic evidence that the *NtIAA* family may serve as candidate genes in response to dicamba, offering potential genetic candidates for breeding herbicide-resistant crops.

## Introduction

Modern agricultural production still relies heavily on chemical herbicides. Dicamba (3,6-dichloro-2-methoxybenzoic acid), a highly effective synthetic auxin herbicide, is widely used in agriculture due to its low toxicity, high selectivity, and exceptional efficacy against broadleaf weeds (Behrens et al., 2007; Wei et al., 2025). However, field application of this herbicide is associated with drift and residue issues, which cause adverse effects on non-target crops, including leaf curling, growth retardation, and photosynthetic disruption (Pan et al., 2022). Furthermore, the long-term and extensive use of similar herbicides not only promotes rapid evolution of herbicide resistance in weed populations but also restricts existing crop rotation systems due to herbicide residues, thereby negatively impacting the sustainable development of agricultural ecosystems (Pan et al., 2022; Tang et al., 2025). Therefore, in-depth exploration of molecular response mechanisms of crops to dicamba stress is crucial for reducing herbicide damage and ensuring crop safety in production, while also advancing green and sustainable agriculture development.

With the rapid advancement of genome editing and genetic engineering technologies, CRISPR/Cas9-mediated genome editing has provided strategies for developing crop germplasms with specific resistance traits. However, most current research focuses on disease and pest resistance breeding, while studies on herbicide resistance trait development remain relatively limited. Currently, genome editing of the rice *OsAFB4* gene has conferred cross-resistance to two distinct synthetic auxin herbicides, dicamba and picloram, without incurring significant grain yield loss (Wei et al., 2025). Further studies have elucidated the functional specificity and redundancy among members of the OsTIR1/AFB receptor family in mediating herbicide responses (Guo et al., 2021). In addition to target-site modification approaches, studies on non-target-site resistance mechanisms have revealed that promoter methylation of cytochrome P450 monooxygenase family members (e.g., *CYP81A68*) regulates rice resistance to multiple herbicides. Moreover, MATE family proteins (e.g., DTX6) can effectively reduce intracellular herbicide concentration by compartmentalizing herbicide molecules into vacuoles or extruding them outside the cell (Lv et al., 2021). These findings collectively underpin both the mechanistic understanding of herbicide resistance and the molecular design of herbicide-resistant cultivars.

As a synthetic auxin herbicide, dicamba disrupts plant growth by interfering with hormone-regulated processes (Behrens et al., 2007; Yao et al., 2016). The Dmt methyltransferase from *Sphingomonas* sp. inactivates dicamba via a tetrahydrofolate-dependent demethylation, and its heterologous expression confers herbicide resistance in plants. Tobacco is an ideal model plant for studying stress responses, with its well-annotated genome (Edwards et al., 2017) and genetic transformation system (Cody et al., 2023) providing a foundation for transcriptomic research. Herbicide resistance has been extensively studied in several crops, including rice (Dong et al., 2024; Li et al., 2025; Ouyang et al., 2025), rapeseed (Zhang et al., 2025; Liu et al., 2025), and sorghum (Tang et al., 2025). In contrast, the transcriptional response of tobacco to dicamba stress-especially with respect to auxin-related pathway remodeling and transporter regulation-remains largely unexplored.

This study uses high-throughput transcriptome sequencing to profile genome-wide expression dynamics in tobacco leaves under dicamba stress across multiple time points. Screening differentially expressed genes (DEGs) and regulatory networks, we aim to identify dicamba-modulated pathways and predict functional modules and transcription factors for detoxification and tolerance. These findings offer a descriptive basis for understanding dicamba phytotoxicity in tobacco and identifying candidate genes for herbicide-resistant breeding.

## Materials and methods

### Plant material and treatments

*Nicotiana tabacum* cv. K326 seeds were surface-sterilized by immersion in 10% sodium hypochlorite solution for 15 minutes, followed by five rinses with distilled water. The sterilized seeds were sown onto Murashige and Skoog (MS) agar medium plates and incubated in an artificial climate chamber under controlled conditions: 16h light at 30°C and 8h darkness at 26°C, with 60% relative humidity. When seedlings developed their first true leaves, they were treated with 50μM dicamba (Sigma) applied as 10 mL of aqueous solution directly to each plate. As dicamba is a systemic herbicide, this method allowed root uptake and subsequent translocation. To profile the dynamic transcriptional response, shoots (including stems and leaves) were harvested at four time points: untreated control, and 6h, 24h, and 72h post-treatment. At each time point, three independent biological replicates were collected, each consisting of a pooled sample from 10 seedlings. Before each sampling, visible symptoms (e.g., root growth inhibition) were observed. Immediately after collection, all samples were flash-frozen in liquid nitrogen and stored at -80°C until further analysis.

### RNA extraction, library construction, and sequencing

Total RNA was extracted for library preparation. mRNA enrichment was performed using poly-T oligo-attached magnetic beads, followed by cDNA synthesis. Briefly, first-strand cDNA was synthesized using random hexamer primers and M-MuLV Reverse Transcriptase (RNase H-). Second-strand cDNA synthesis was then catalyzed by DNA Polymerase I and RNase H. The cDNA was fragmented, and the AMpure XP system (Beckman Coulter, Beverly, USA) was used to select fragments of 370-420 bp. After adaptor ligation, PCR amplification was performed to complete the library. Sequencing was conducted on the Illumina NovaSeq platform, generating 150 bp paired-end reads.

### Transcript assembly and functional annotation

Quality control of raw sequencing data was performed using Fastp (v0.19.7) (Chen et al., 2018). Clean reads were obtained by filtering as follows: (1) removal of read pairs containing adapter sequences; (2) exclusion of read pairs with >10% of ambiguous bases (N); and (3) discarding read pairs with > 50% of bases having a Phred quality score <5. The clean reads were then aligned to the K326 reference genome (Edwards et al., 2017) using HISAT2 (v2.1.0) (Kim et al., 2019). Finally, transcripts for each sample were reconstructed with StringTie (Pertea et al., 2015). For functional annotation, Gene Ontology (GO) terms were assigned using PANNZER2 (Törönen et al., 2018), while KEGG pathway mapping was conducted via BlastKOALA (Kanehisa et al., 2016).

### Differential gene screening and enrichment analysis

Gene expression levels were quantified and normalized using the RPKM (Reads Per Kilobase per Million mapped reads) method implemented in StringTie. Differential expression analysis was performed with DESeq2 v1.20.0 (Love et al., 2014), and DEGs were identified using thresholds of false discovery rate (FDR) < 0.05 and |log2(fold change)| > 1.

GO (Gene Ontology) and KEGG (Kyoto Encyclopedia of Genes and Genomes) enrichment analyses were carried out with TBtools-II (v2.371). For both analyses, statistical significance was assessed using the hypergeometric test (Fisher’s exact test), *p*-values were adjusted for multiple comparisons using the Benjamini-Hochberg FDR (false discovery rate) method with an adjusted *p*-value < 0.05 consider significant. Transcription factor (TF) enrichment analysis was conducted using the hypergeometric test with the same FDR correction (adjusted *p* < 0.05). All enrichment results were visualized using an online bioinformatics platform (https://www.bioinformatics.com.cn/).

### Weighted gene co-expression network analysis

WGCNA was performed using OECloud tools (https://cloud.oebiotech.com) with default parameters. Genes with low expression variation (SD ≤ 0.5) were filtered out, leaving 8,377 genes for subsequent analysis. Based on scale-free topology criterion and mean connectivity, the soft-thresholding power was set to 30 for constructing the adjacency matrix. The correlation between module eigengenes and sample traits were evaluated in R (v4.5.1). Modules with a Pearson’s correlation coefficient *r* > 0.6 and *p*-value < 0.01 were identified as significantly associated with dicamba treatment. Within these modules, the top 50 genes with highest connectivity were extracted to construct co-expression networks using Cytoscape (v3.10.4) (Shannon et al., 2003). Hub genes were identified as those with highest degree centrality in each network. Functional interpretation of modules was explored via GO and KEGG enrichment analyses.

### Phylogenetic and protein domain analysis

The AUX/IAA (NtIAA) protein sequences were obtained from genomic databases: *Arabidopsis thaliana* sequences from TAIR (https://www.arabidopsis.org/) (Reiser et al., 2024), *Oryza sativa* sequences from Phytozome (https://phytozome-next.jgi.doe.gov/) (Yuan et al., 2003), and *Nicotiana tabacum* sequences from Solanaceae Genomics Network (SGN, https://solgenomics.net/) (Edwards et al., 2017). A phylogenetic tree of NtIAA proteins from the three species was constructed using the Neighbor-Joining method with 1000 bootstrap replicates in MEGA11 (Tamura et al., 2021). The resulting Newick file was uploaded to iTOL (https://itol.embl.de/) (Letunic and Bork, 2021) for tree visualization. Conserved motifs in NtIAA proteins were predicted using the Simple MEME Wrapper module of TBtools, with motif discovery number set to 10 and other parameters at default. To validate domains, tobacco NtIAA sequences were submitted to NCBI Conserved Domain Database (CDD, https://www.ncbi.nlm.nih.gov/Structure/cdd/wrpsb.cgi) (Wang et al., 2023) for annotation. The conserved motifs and CDD predictions were visualized using the Gene Structure View (Advanced) function in TBtools.

### Gene synteny analysis and characterization

Genomic sequences and annotation files for *Arabidopsis thaliana* and *Nicotiana attenuata* (Xu et al., 2017) were obtained from EnsemblPlants (https://plants.ensembl.org/index.html). Data for *Nicotiana tabacum* and *Nicotiana benthamiana* (Bombarely et al., 2012) were obtained from Solanaceae Genomics Network (SGN), and for rice (*Oryza sativa*) from Phytozome. Genomic resources for *Nicotiana tomentosiformis* and *Nicotiana sylvestris* (Sierro et al., 2024) were obtained from NCBI (https://www.ncbi.nlm.nih.gov/). Synteny analysis was conducted using the One-Step MCScanX Super-Fast plugin in TBtools with default parameters, and results were visualized using the Multiple Synteny Plot module.

### Cis-acting element analysis of *NtIAA*

The 2,000-bp promoter regions immediately upstream of the transcription start sites of *NtIAA* were extracted from the genome via the Gtf/Gff3 Sequences Extract function of TBtools. Putative cis-regulatory elements were subsequently predicted by submitting these promoter sequences to the PlantCARE online database (https://bioinformatics.psb.ugent.be/webtools/plantcare/html/) (Lescot et al., 2002). To ensure data reliability, the initial predictions were manually curated to remove irrelevant and uncharacterized elements. The final set of curated cis-regulatory elements was visualized using the Simple BioSequence Viewer module in TBtools.

### Transcription factor binding site prediction

Prediction was performed using the online “Binding Site Prediction” function based on the PlantRegMap database (https://plantregmap.gao-lab.org/) (Tian et al., 2020). The promoter sequences or genomic coordinates of the target genes were first input, with statistical significance thresholds set (*p*-value ≤ 1×10^-6^, *q*-value < 0.05). The platform then performed matching calculations based on its compiled plant transcription factor binding profile data and outputted binding sites and associated transcription factors that met the significance criteria. Finally, the regulatory relationships between the screened transcription factors and candidate genes were used to construct a visual network diagram in Cytoscape software. The correlation analysis between transcription factors and *NtIAA* was performed using the Pearson correlation coefficient.

### Quantitative real-time PCR

Primers were designed with Primer3 tool (Untergasser et al., 2012). The *L25* gene was used as the reference gene. All primer sequences were included in **Supplementary Table 2**. Total RNA was obtained with a plant RNA rapid extraction kit (Genepure Plus, Imagene, China). First-strand cDNA was synthesized with AMV reverse transcriptase and oligo dT primer (Takara Bio Inc., Dalian, China). qRT-PCR was performed in a reaction volume of 20 μl containing 1 μl of specific primers (10 μM), 10μl of the SYBR Green qPCR mix, 2 μl of the template cDNA (50 ng/μl), and 7 μl of ddH_2_O. qPCR analysis was carried out using a CFX96™ Real-Time System (Bio-Rad, Hercules, CA, United States). At each time point, RNA was extracted from a pool of five seedlings and subjected to three technical replicates. Relative expression levels were calculated using the 2^-ΔΔCt^ method, and data are presented as mean ± standard error (SE). An *F*-test was first performed to assess the homogeneity of variances, followed by a *t*-test to compare the significance of differences between each treatment time point and the control.

## Results

### Quality control of transcriptome data

**Figure 1:**
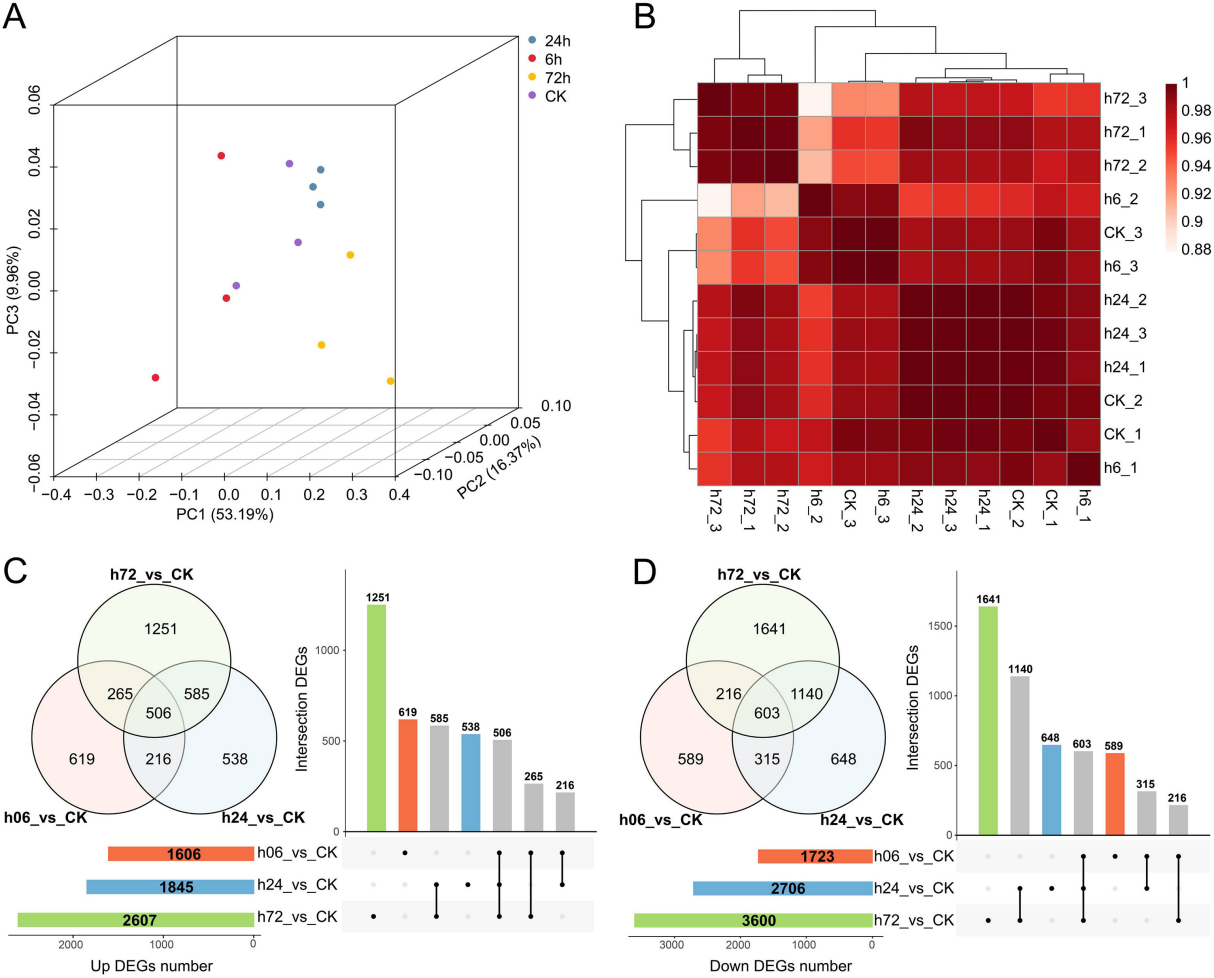
Overview of transcriptomic data analysis. **(A)** Principal component analysis (PCA). The x-, y-, and z-axes correspond to PC1, PC2, and PC3 scores, respectively. Each point represents an individual sample, with colors indicating different treatment time points (Control, 6h, 24h, 72h). **(B)** Inter-sample correlation analysis. The heatmap displays Pearson correlation coefficients between all sample pairs. Color intensity reflects the magnitude of correlation coefficients (darker red indicates higher correlation). **(C)** Quantification of upregulated DEGs. The Upset plot displays the total number of DEGs significantly upregulated across the three comparison groups. Isolated solid dots denote DEGs uniquely identified at single time points, while the Venn diagram summarizes the counts of unique and shared DEGs among groups. **(D)** Quantification of downregulated DEGs. The upset plot displays the total number of significantly downregulated DEGs across the three comparison groups. Isolated solid dots represent DEGs specifically downregulated at individual time points, while the venn diagram summarizes the counts of unique and shared DEGs among groups.

To profile transcriptome changes across different time points (CK, 6h, 24h, and 72h), 12 samples were sequenced, generating 200.08 Gb of raw data (**Supplementary Table 1**). After removing low-quality sequences, clean reads per sample ranged from 100.71 to 152.18 million (average ~16.48 Gb). GC content remained stable (41.01%-41.52%), and Q30 scores exceeded 91.31%, confirming sufficient data quality for expression analyses. Principal component analysis (PCA) showed distinct separations among time points and clustering of biological replicates (**Figure 1A**). Sample correlation analysis further demonstrated highly consistent expression patterns within each group (**Figure 1B**). These results indicate that the transcriptomic data exhibit good reproducibility and reliability, supporting their suitability for in-depth analyses.

### Global transcriptional changes under dicamba stress

Comparative analysis across different time points after dicamba treatment revealed a consistent increase in upregulated DEGs compared to the control, rising from 1,606 at 6h to 1,845 at 24h, and further to 2,607 at 72h (**Figure 1C**). Similarly, downregulated DEGs showed a marked upward trend, increasing from 1,723 at 6h to 2,706 at 24h, and finally to 3,600 at 72h (**Figure 1D**). Additionally, a set of 506 consistently upregulated DEGs (**Figure 1C**) and 603 consistently downregulated DEGs (**Figure 1D**) across all time points was identified.

### Pathway-level responses revealed by GO and KEGG enrichment

#### GO functional enrichment analysis of DEGs

**Figure 2:**
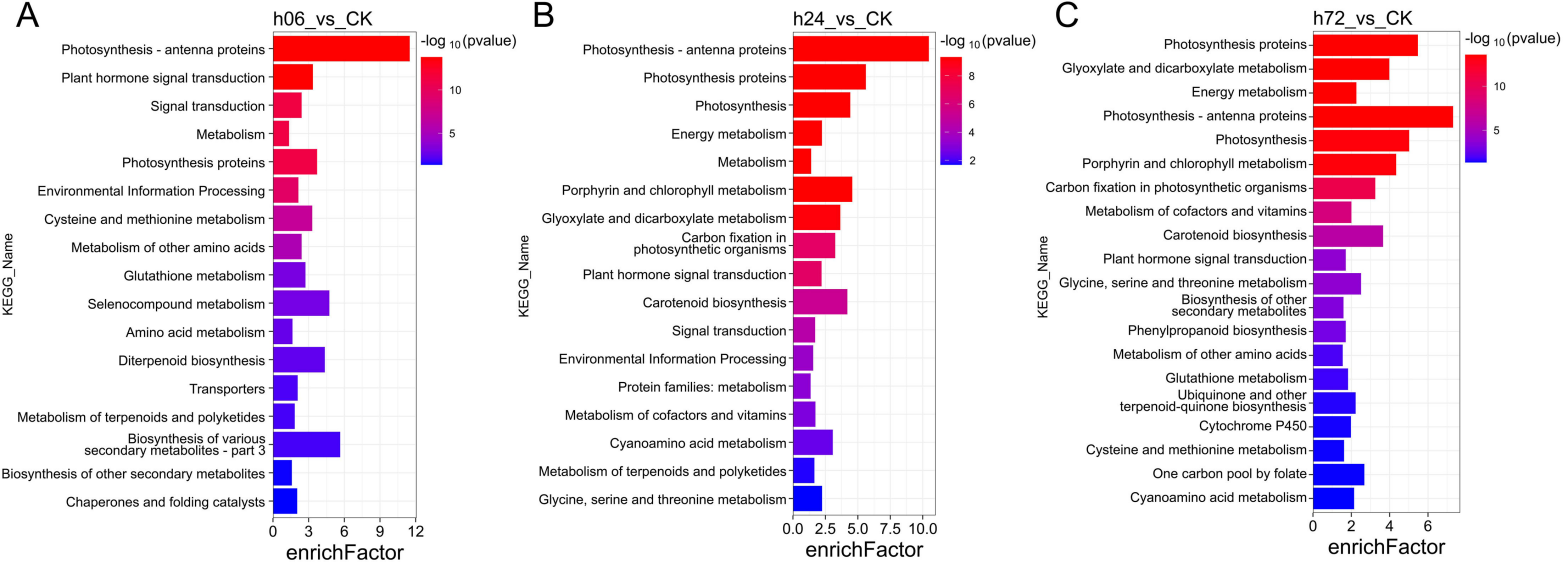
GO enrichment analysis of DEGs at different time points after dicamba treatment. **(A)** GO enrichment terms for DEGs screened from the comparison between the 6-hour treatment group and the control group. **(B)** GO enrichment terms for DEGs screened from the comparison between the 24-hour treatment group and the control group. **(C)** GO enrichment terms for DEGs screened from the comparison between the 72-hour treatment group and the control group. In each panel, the dot size indicates the number of genes enriched in the pathway, and the color scale from blue to red represents the significance level (adjusted *p*-value).

GO enrichment analysis of DEGs at different time points after dicamba treatment suggested a progressively deepening response pattern at the molecular level. At the early stage (6h), methionine metabolic reprogramming and photosystem I response were initiated, accompanied by activation of methyltransferases and histidine kinases, potentially contributing to stress perception and signal transduction (**Figure 2A**). During the intermediate stage (24h), the response may have shifted toward photosynthetic structural remodeling and oxidative defense, possibly accompanied by sustained tetrapyrrole metabolism, as well as adjustments in photosystem I/II and thylakoid membrane components (**Figure 2B**). In the late stage (72h), the focus could have moved toward the repair and assembly of photosystem II, the optimization of the photosynthetic electron transport chain, and the coordination of carbon fixation pathways (**Figure 2C**). It is also possible that energy homeostasis might be reestablished through the maintenance of tetrapyrrole metabolism and related enzyme activities.

### KEGG functional enrichment analysis of DEGs

**Figure 3:**
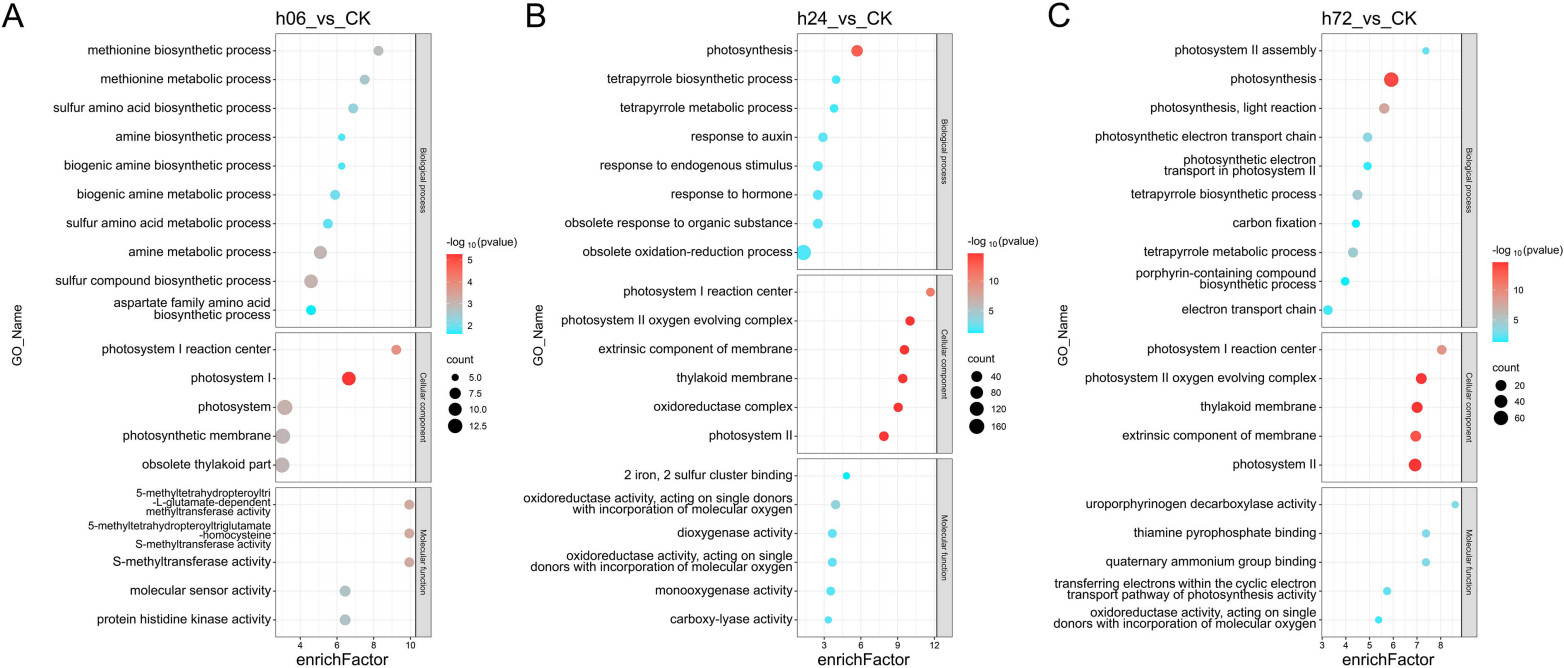
KEGG enrichment analysis of DEGs at different time points after dicamba treatment. **(A)** KEGG enrichment terms for DEGs screened from the comparison between the 6-hour treatment group and the control group. **(B)** KEGG enrichment terms for DEGs screened from the comparison between the 24-hour treatment group and the control group. **(C)** KEGG enrichment terms for DEGs screened from the comparison between the 72-hour treatment group and the control group. The bar length indicates the enrichment factor (ratio of observed to expected gene counts), and the color scale from blue to red represents the significance level (adjusted *p*-value).

Under dicamba stress, KEGG enrichment analysis of DEGs would suggest a time-dependent response in plant metabolic pathways, though these results remain hypothetical. At the early stage (6h), the response might be primarily characterized by amino acid metabolism and antioxidant systems (cysteine-methionine and glutathione metabolism), possibly accompanied by an initial activation of hormone signaling pathways (**Figure 3A**). During the intermediate stage (24h), photosynthesis-related pathways (antenna proteins, chlorophyll metabolism, carbon fixation) could become enriched, while photoprotective mechanisms such as carotenoid biosynthesis might simultaneously be enhanced (**Figure 3B**). By the late stage (72h), although photosynthetic pathways would still appear active, secondary metabolic pathways including one-carbon metabolism, ubiquinone synthesis, and phenylpropanoid metabolism might be significantly activated, potentially forming a multi-layered defense-repair network (**Figure 3C**). This hypothetical response trend would be tentatively consistent with the GO enrichment results.

### Identification of co-expression modules and candidate hub genes via WGCNA

**Figure 4:**
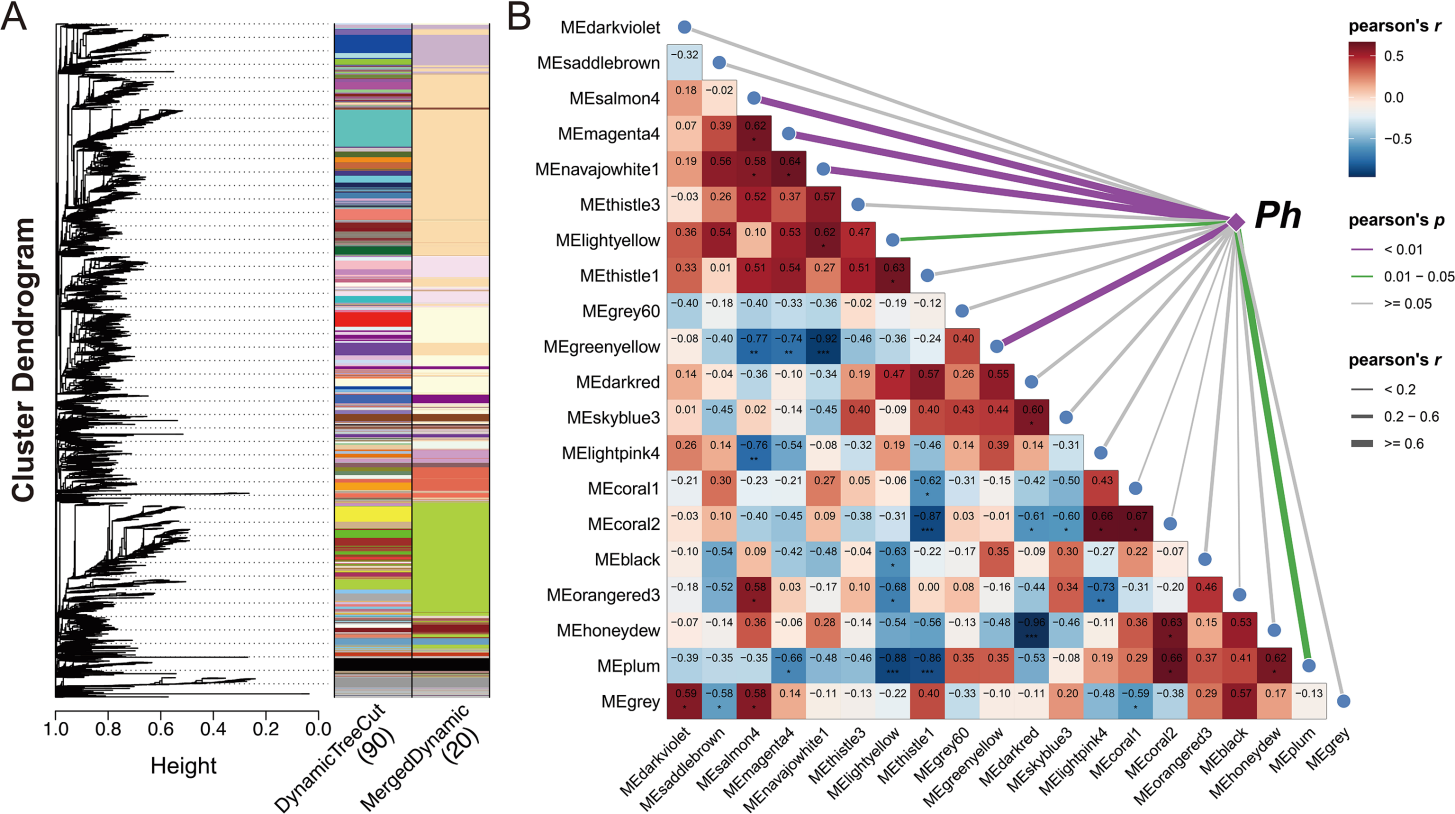
Co-expression network analysis identifies gene modules associated with dicamba treatment. **(A)** Hierarchical clustering dendrogram of genes. The dendrogram was constructed using WGCNA based on topological overlap dissimilarity. Each colored row below the dendrogram represents a distinct co-expression module, with a total of 20 modules identified. The color is shown at the top. **(B)** Module-trait association heatmap. The heatmap displays correlation coefficients between module eigengenes and dicamba treatment time points (Control, 6h, 24h, 72h). Red indicates positive correlation, blue indicates negative correlation, and color intensity reflects the correlation strength. Asterisks denote statistical significance (*p < 0.05, **p < 0.01, ***p < 0.001). For the network diagram inset (if applicable), line width corresponds to Pearson’s correlation coefficient (thicker lines indicate stronger correlations), and line color represents the significance level (darker colors indicate higher significance). “Ph” in this paper represents the treatment and is used as an abbreviation for phenotype.

To further explore the gene regulatory network in tobacco under dicamba treatment, WGCNA (weighted gene co-expression network analysis) was employed to partition all 8,377 genes into 20 co-expression modules under the predetermined network construction parameter (power value = 30) (scale-free topology plot and justification are provided in **Supplementary Figures 1A–D**) (**Figure 4A**). Through correlation analysis between module eigengenes and different treatment time points, we identified four modules significantly associated with treatment duration: the Salmon4 (69 genes), Magenta4 (150 genes), navajowhite1 (2,799 genes), and greenyellow (1,552 genes) (**Figure 4B**).

Functional annotation revealed that: the greenyellow module was primarily associated with photosynthesis and tetrapyrrole metabolism (**Supplementary Figures 2A, B**); the navajowhite1 module was mainly enriched in plant hormone signal transduction, amino acid metabolism, and energy metabolism (**Supplementary Figures 2C, D**); while both Salmon4 and Magenta4 modules were co-enriched in molecular binding and basic metabolic pathways (**Supplementary Figures 2E, F**). Furthermore, gene interaction networks were constructed for these modules by selecting the top 50 genes based on internal connectivity of the module. The results revealed that the Salmon4, Magenta4, navajowhite1, and greenyellow modules contained 3 (*Nitab4.50001903g0130*, *Nitab4.50000004g0150*, *Nitab4.50004994g0040*) (**Supplementary Figure 3A**), 3 (*Nitab4.50002836g0020*, *Nitab4.50011688g0010*, *Nitab4.50011467g0020*) (**Supplementary Figure 3B**), 1 (*Nitab4.50004287g0050*) (**Supplementary Figure 3C**), and 4 (*Nitab4.50000543g0120*, *Nitab4.50006395g0010*, *Nitab4.50000119g0130*, *Nitab4.50013317g001*0) (**Supplementary Figure 3D**) candidate hub genes, respectively, which may play important roles in maintaining module network structure (**Supplementary Figure 3**).

### Identification and functional analysis of NtIAA transcription factors

**Figure 5:**
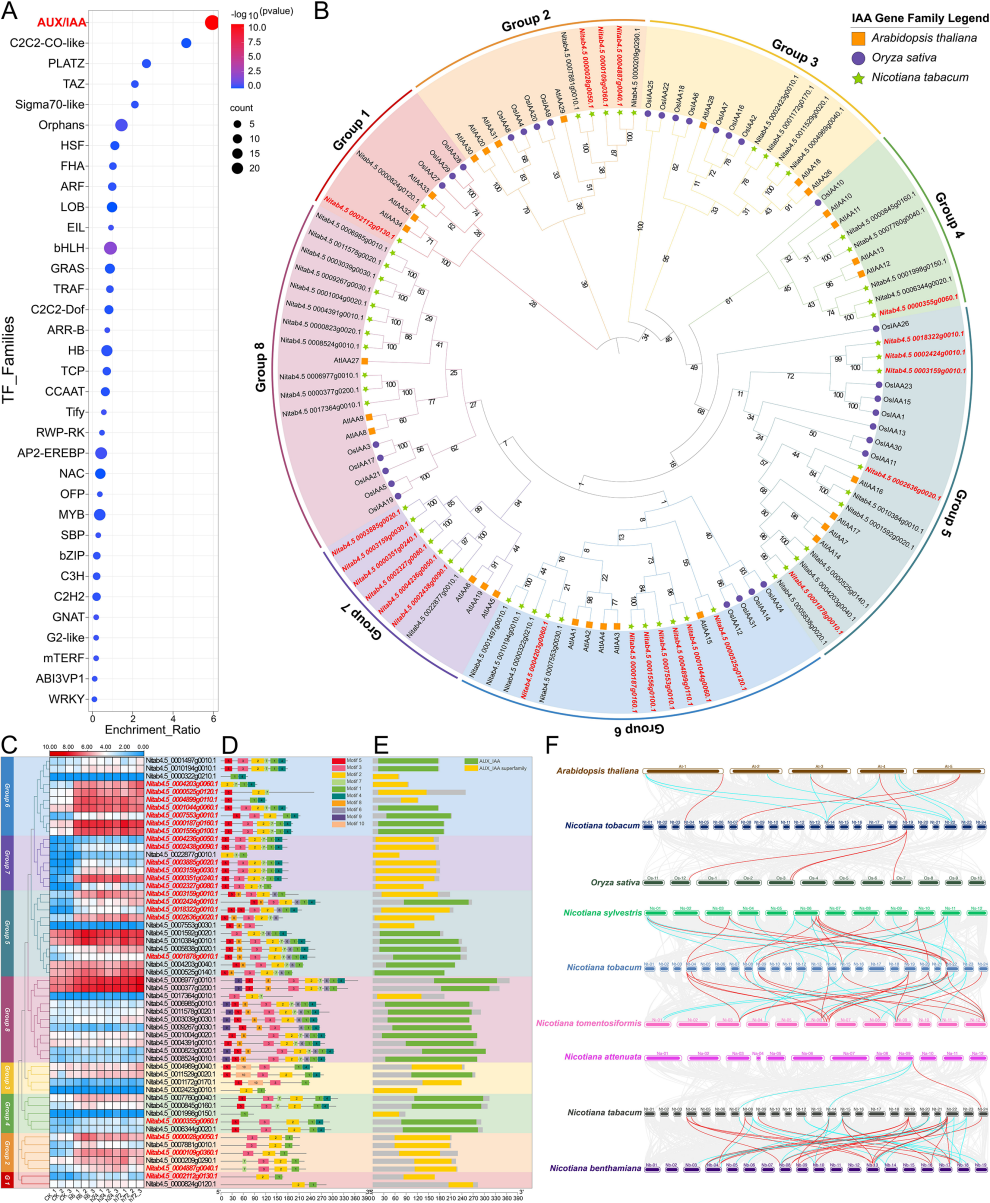
Comprehensive analysis of NtIAA transcription factors in response to dicamba treatment. **(A)** Transcription factor enrichment analysis. Dot size corresponds to the number of enriched genes, while the color gradient (blue to red) represents the statistical significance of enrichment (-log_10_(*p*-value)). **(B)** Phylogenetic analysis of NtIAA proteins from tobacco, *Arabidopsis*, and rice. Proteins highlighted in red represent differentially expressed transcription factors (DE-TFs).Bootstrap values are indicated at branch nodes. **(C)** Temporal expression patterns of *NtIAA* genes. The heatmap displays expression levels across different time points, with red and blue indicating high and low expression, respectively. **(D)** Conserved motif composition of NtIAA proteins. Distinct motifs are represented by color-coded boxes and numbered 1-10. **(E)** Domain architecture of NtIAA proteins. Different protein domains are depicted in distinct colors. **(F)** Synteny analysis of *NtIAA* genes across species. The analysis includes model plants (*Arabidopsis thaliana* and *Oryza sativa*), ancestral tobacco species (*Nicotiana tomentosiformis* and *Nicotiana sylvestris*), and other *Nicotiana* species (*Nicotiana attenuata* and *Nicotiana benthamiana*). Blue lines indicate non-differentially expressed *NtIAA* genes, while red lines denote differentially expressed ones.

Among the common differentially expressed genes identified across different time points following dicamba treatment, 155 encode proteins belonging to transcription factor families. Enrichment analysis of various transcription factor types revealed significant over-representation of the NtIAA (23 members) and bHLH (15 members) families (**Figure 5A**). This is consistent with dicamba’s mode of action as a synthetic auxin herbicide (Behrens et al., 2007).

Phylogenetic analysis of all NtIAA proteins in the tobacco genome revealed that 54 members were classified into eight subgroups. Among these, 23 differentially expressed *NtIAA* were distributed across six subgroups: Group 1 (1), 2 (3), 4 (1), 5 (5), 6 (7), and 7 (6) (**Figure 5B**). All these *NtIAA* members exhibited upregulation following dicamba treatment (**Figure 5C**), suggesting that the transcriptional regulatory system within the auxin signaling pathway may remain persistently activated in tobacco under dicamba stress. Analysis of conserved motifs and domains revealed that NtIAA proteins commonly contain important motifs (Motif 1–5 and 7) and the AUX_IAA domain (**Figures 5D, E**). Group 8 and Group 3 uniquely possess Motif 9 and Motif 10, respectively. The absence of differential expression in these subgroups suggests potential roles in subfamily-specific functional regulation. Collinearity analysis further revealed that tobacco exhibits a closer evolutionary relationship with the dicot *Arabidopsis* (13 homologous gene pairs), than with the monocot rice (4 pairs). Notably, tobacco and *N. benthamiana* share the highest number of homologous gene pairs (52 pairs), significantly exceeding those with its ancestral species *N. tomentosiformis* (38 pairs) and *N. sylvestris* (34 pairs), as well as other related species such as *N. attenuata* (5 pairs) (**Figure 5F**). These findings suggest that the *NtIAA* gene family may have undergone lineage-specific expansion or retention, while also experiencing differential evolutionary selection among distinct *Nicotiana* species.

### Cis-elements prediction of *NtIAA* genes

**Figure 6:**
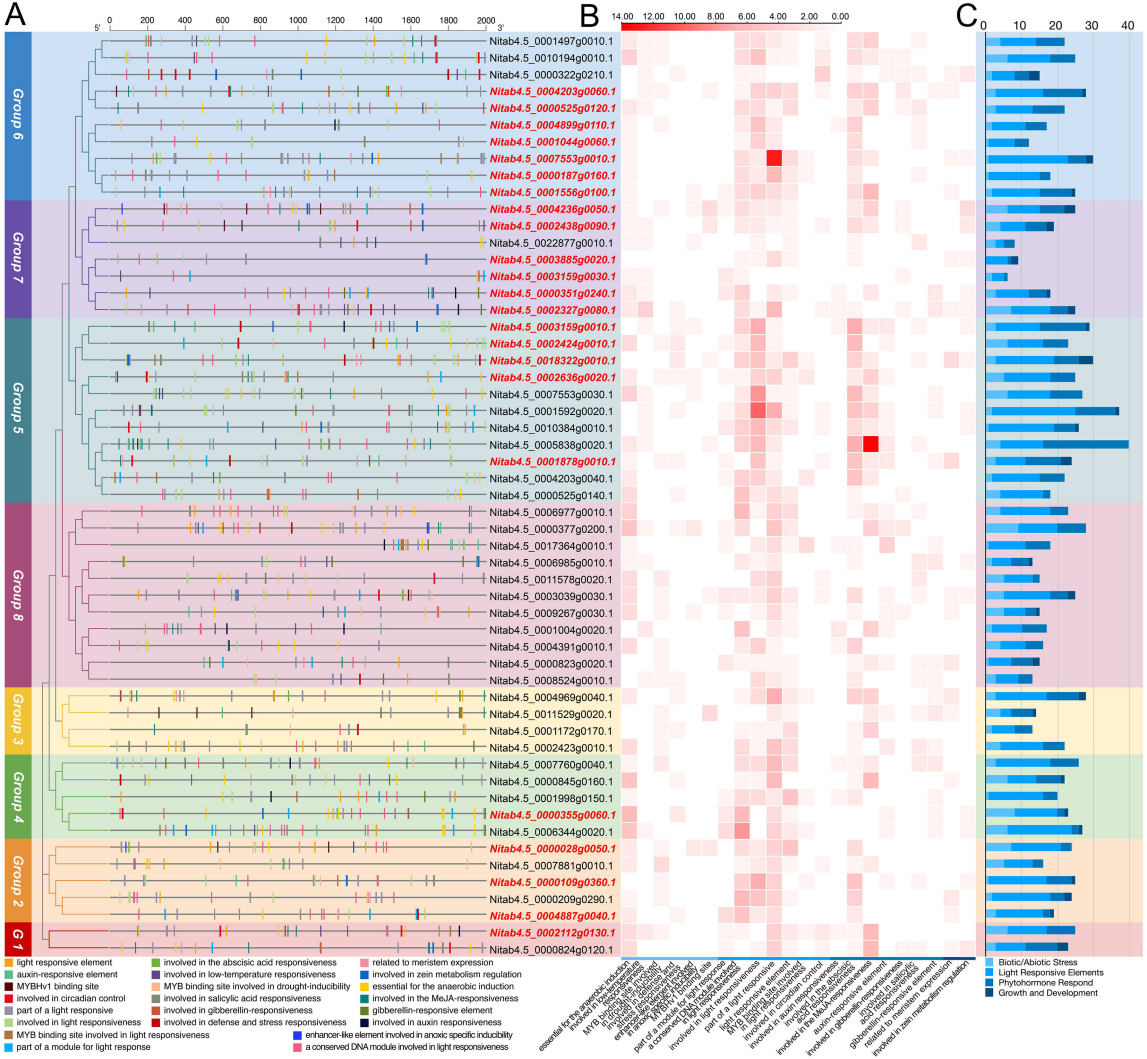
Analysis of cis-acting elements in the 2000-bp promoter region upstream of *NtIAA* genes. **(A)** Distribution profile of cis-acting elements across promoter sequences. Distinct element types are represented by color-coded boxes. **(B)** Quantitative analysis of cis-acting elements per gene, with color intensity reflecting element abundance (darker red indicates higher counts). **(C)** Stacked bar plot showing total cis-acting elements per gene and their distribution across functional subgroups.

In-depth analysis of *NtIAA* gene promoter sequences identified 22 categories of cis-acting elements (**Figures 6A, B**), classified into four distinct functional groups (**Figure 6C**). Light-responsive and phytohormone-responsive elements exhibited the highest diversity (7 types each) and dominated in abundance (**Figures 6A–C**). The distribution of these elements across promoters showed significant heterogeneity. For instance, the copy number of MeJA-responsive elements reached 14 in the promoter of *Nitab4.5_0005838g0020*, whereas other family members contained no more than 6 copies (**Figures 6A, B**). Comparative analysis of functional categories further revealed distinct distribution patterns among different element types.

### Construction of the predictive regulatory network and screening of candidate genes

**Figure 7:**
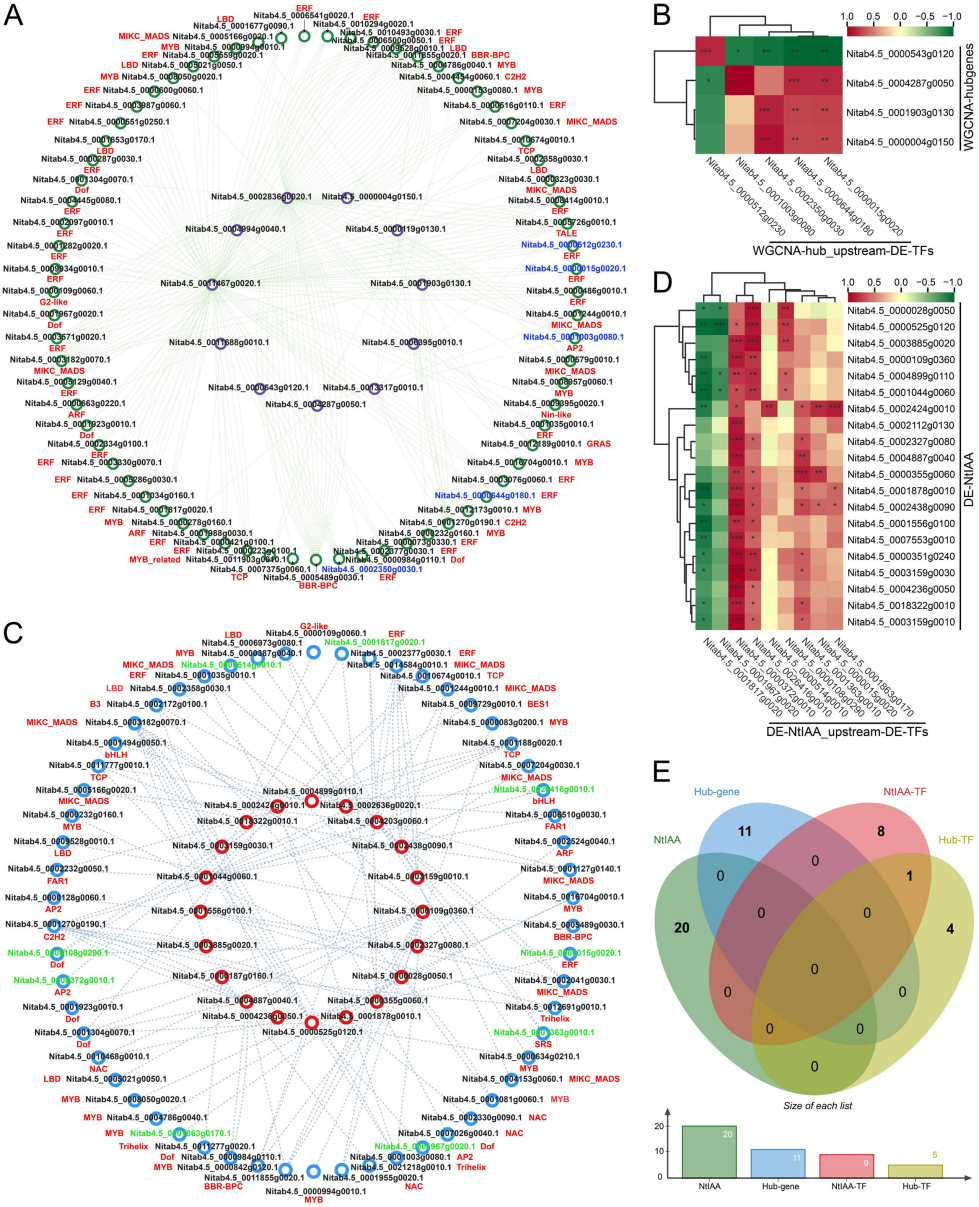
Predictive regulatory network of tobacco in response to dicamba stress. **(A)** Predictive regulatory network of module hub genes and their upstream transcription factors in tobacco under dicamba stress. The purple inner circle represents module hub genes identified by WGCNA, while the green outer circle shows the predicted upstream transcription factors regulating these hub genes. Genes marked in blue indicate those highly correlated with the module hub genes. Node size is proportional to connectivity. Edge colors (gray to black) represent the strength of correlation or prediction confidence. Important note: All connections (edges) between nodes in this figure are inferred based on expression correlation analysis and promoter cis-element prediction (e.g., using PlantRegMap or similar tools). They represent computational predictions, not experimentally validated interactions. **(B)** Correlation heatmap between module hub genes and their upstream transcription factors. Abbreviations used in this figure: WGCNA-hub genes, differentially expressed hub genes identified by WGCNA; WGCNA-hub_upstream-DE-TFs, differentially expressed transcription factors predicted to regulate the WGCNA-identified hub genes. Color intensity reflects the magnitude of the correlation coefficient. Asterisks indicate statistical significance levels (*p < 0.05, **p < 0.01, ***p < 0.001). **(C)** Predictive regulatory network of *DE-NtIAAs* and their upstream transcription factors. The red inner circle represents *DE-NtIAAs*, and the blue outer circle represents the predicted upstream transcription factors regulating *NtIAA* expression. Genes labeled in green show high correlation with *DE-NtIAAs*. Node size and edge styling follow the same conventions as in **(A)**. **(D)** Correlation heatmap between DE-*NtIAAs* and their upstream transcription factors. Abbreviations used in this figure: DE-*NtIAA*, differentially expressed *NtIAA* genes; DE-*NtIAA*_upstream-DE-TFs, differentially expressed transcription factors predicted to regulate the differentially expressed *NtIAA* genes. Color intensity reflects the magnitude of the correlation coefficient. Asterisks indicate statistical significance levels (*p < 0.05, **p < 0.01, ***p < 0.001). **(E)** Venn diagram showing unique and overlapping gene counts among four different gene sets. (e.g., hub genes, DE-*NtIAAs*, and their predicted TFs). Each circle corresponds to one gene set, and overlapping regions indicate shared genes.

In this study, we performed predictive analysis on upstream regulatory transcription factors of the 11 hub genes identified by WGCNA, identifying 71 genes from 15 transcription factor families. Among these, 15 were DEGs, including ERF (13), Dof (1), and AP2 (1) families (**Figure 7A**). Correlation analysis demonstrated that among these 15 DEGs, 5 transcription factors (4 positively correlated and 1 negatively correlated) exhibited significant associations with the expression of 4 candidate genes (**Figure 7B**).

Concurrently, upstream regulatory transcription factor prediction was performed for the *NtIAA* gene family. A total of 59 potential regulatory genes were identified, belonging to 18 transcription factor families, of which 18 were DEGs, categorized into AP2 (3), MYB (3), bHLH (1), Dof (3), MIKC_MADS (3), ERF (2), TCP (1), SRS (1), and NAC (1) families (**Figure 7C**). Correlation analysis revealed that among these 18 DEGs, 9 transcription factors (2 positively correlated and 7 negatively correlated) showed significant associations with the expression of 20 *NtIAA* genes (**Figure 7D**).

Further integrated analysis revealed that the ERF family member *Nitab4.5_0000015g0020* was predicted to simultaneously bind to *Nitab4.5_0000355g0060* (encoding the NtIAA-ARF dimerization domain) and *Nitab4.5_0011467g0020* (encoding ribosomal protein L27e) (**Figure 7E**). These findings suggest that this ERF transcription factor could be functionally important in the response to dicamba stress. However, the proposed regulatory interactions are currently derived from computational predictions and await experimental confirmation.

### qRT-PCR validation of representative candidate genes

**Figure 8:**
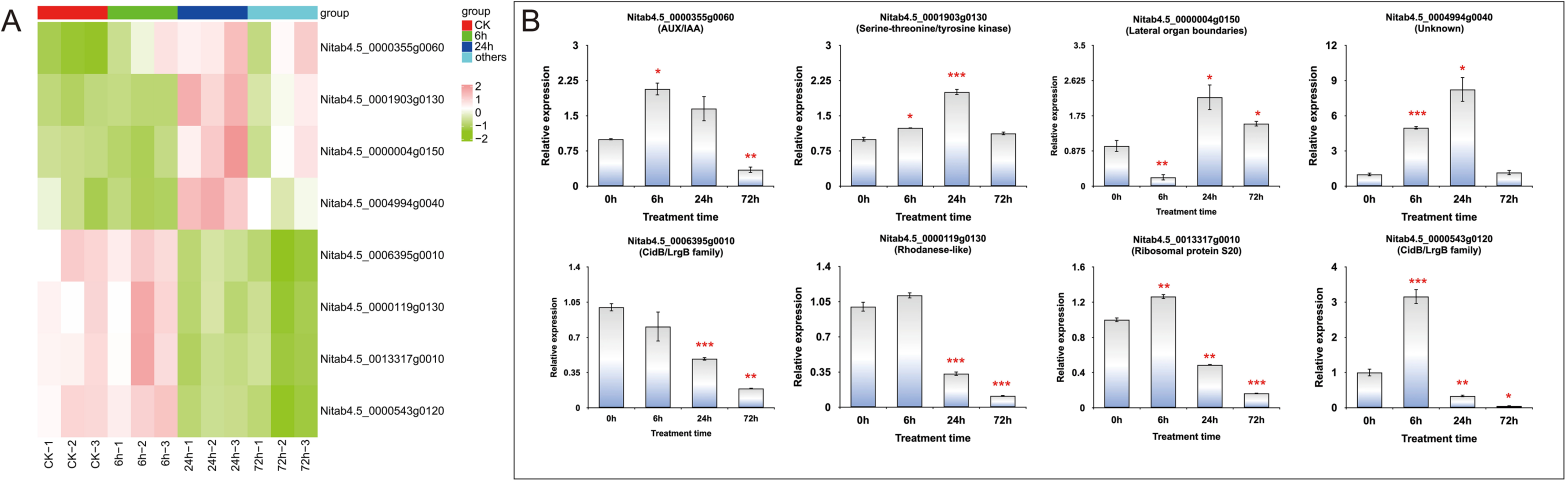
Consistency validation of candidate gene expression between transcriptomic data and qRT-PCR experiments. **(A)** Heatmap of the expression profiles of 8 candidate genes. Rows represent genes, columns represent time points, and color intensity indicates expression level (red: upregulation, blue: downregulation). **(B)** qRT-PCR validation of selected candidate genes. The expression levels of candidate genes *Nitab4.5_0000355g0060* (*NtIAA*), *Nitab4.5_0001903g0130* (Serine-threonine/tyrosine kinase), *Nitab4.5_0000004g0150* (Lateral organ boundaries), *Nitab4.5_0004994g0040* (Unknown), *Nitab4.5_0006395g0010* (CidB/LrgB family), *Nitab4.5_0000119g0130* (Rhodanese-like), *Nitab4.5_0013317g0010* (Ribosomal protein S20), *Nitab4.5_0000543g0120* (CidB/LrgB family) were validated using qRT-PCR. (*p < 0.05, **p < 0.01, ***p < 0.001).

To assess the reliability of the transcriptome sequencing results, we selected 8 representative candidate genes for qRT-PCR validation based on multiple screening strategies (**Figure 8A**). The selected genes represent critical candidates from multiple analytical modules, including: an auxin-responsive NtIAA (*Nitab4.5_0000355g0060*), a serine/threonine/tyrosine kinase (*Nitab4.5_0001903g0130*), a lateral organ boundaries protein (*Nitab4.5_0000004g0150*), a putative protein (*Nitab4.5_0004994g0040*), two CidB/LrgB family members (*Nitab4.5_0006395g0010* and *Nitab4.5_0000543g0120*), a sulfur metabolism-related rhodanese-like protein (*Nitab4.5_0000119g0130*), and a ribosomal protein S20 (*Nitab4.5_0013317g0010*) (**Figure 8B**). The qRT-PCR results showed that the expression trends of all genes under dicamba treatment were highly consistent with the transcriptome sequencing data (**Figures 8A, B**). These findings support the accuracy of the preliminary transcriptome data, indicating that candidate genes identified through different screening methods exhibit reliable expression patterns. This provides a basis for further functional validation and mechanistic studies.

## Discussion

### The *NtIAA* family as a potential candidate in dicamba response

Previous studies have established dicamba acts as a synthetic auxin herbicide (Behrens et al., 2007). In our transcriptomic data, DEGs were significantly enriched in the “plant hormone signal transduction” pathway, with NtIAA family members showing the highest enrichment among all transcription factor families-consistent with auxin signaling being a primary candidate target of dicamba action. CRISPR/Cas9 editing of the auxin receptor gene *OsAFB4* in rice disrupted excessive auxin responses, generating novel germplasm with cross-resistance to dicamba and picloram (Wei et al., 2025). The widespread upregulation of the *NtIAA* genes observed in this study aligns with these rice findings, suggesting a conserved mechanism of dicamba perception and early signal transduction across species. Thus, *NtIAA* genes may serve as both stress-responsive biomarkers and genetic determinants of tobacco sensitivity to dicamba, offering potential value for resistance breeding.

### A putative temporal regulation of secondary metabolism reprogramming

This study describes the temporal transcriptional landscape of tobacco under dicamba stress, suggesting a possible three-stage “perception-defense-repair” adaptive response. This pattern aligns with general principles of plant abiotic stress responses (Li et al., 2022) but may exhibits specificity to synthetic auxin herbicides. Early-stage (6h) metabolic reprogramming (methionine, glutathione, hormone signaling) reveals that auxin herbicides induce oxidative stress and signaling disruption. Mid-stage (24h) shifts to photosynthetic structural remodeling and photoprotection (e.g., carotenoid synthesis). While consistent with typical repair responses after photosynthetic damage (Groves and Franklin, 2025), our data highlight sustained tetrapyrrole metabolism and thylakoid reconstruction, suggesting a potential regulatory role for photosynthetic metabolism. Late stage (72h) shifts toward systemic repair and broad activation of secondary metabolism (e.g., phenylpropanoid, ubiquinone biosynthesis), possibly reflecting regulatory compensation for metabolic disorders caused by prolonged auxin signaling interference (Figueiredo et al., 2022).hemic.

### The *NtIAA* gene family may serve as putative responsive components in plant responses to dicamba stress

The enrichment of *NtIAA* genes under dicamba stress is linked to their repressive role in auxin signaling (Cao et al., 2019; Thakur et al., 2001). Their ubiquitin-mediated degradation releases ARF activity to initiate downstream gene expression (Kubalová et al., 2025), consistent with the rapid *NtIAA* induction observed in this study, which may represent a compensatory mechanism against excessive auxin-mimicking signals. However, recent evidence indicates that NtIAA degradation is neither sufficient nor necessary for downstream regulation; instead, the AC activity of TIR1 and cAMP play indispensable roles (Chen et al., 2025). The rapid *NtIAA* induction observed may thus result from such multi-level amplification. Evolutionary analysis further reveals functional differentiation among NtIAA members: some likely act as classical rapid-response degradation candidates, while others may form stable inhibitory complexes, helping explain the complex transcriptional response to dicamba.

### Candidate gene prediction and study limitations

Based on computational predictions and expression correlation, we propose a testable hypothesis: the ERF member Nitab4.5_0000015g0020 may be a putative candidate component in tobacco’s response to dicamba stress. It shows sustained upregulation at 24h and 72h (> 6-fold vs. control), and its expression significantly correlated with multiple *NtIAA* genes. Predictive network analysis suggests this ERF might potentially bind two candidate genes: *Nitab4.5_0000355g0060* (auxin signaling)signaling and *Nitab4.5_0011467g0020* (encoding ribosomal protein L27e). Given the known roles of ERF in ethylene signaling and stress responses (Khan et al., 2024), we speculate this gene may act as a predicted linking node between auxin signaling and translation-related processes. Future manipulation of such ERF factors could theoretically be explored for balancing crop tolerance and herbicide efficacy, though this remains highly speculative. We also recognize that this study lacks quantitative phenotypic/physiological data, dose-response analysis, root-specific transcriptomic profiling, and functional validation of candidate genes. Therefore, the current conclusions are hypothesis-generating in nature and require further experimental testing.

## Conclusion

In summary, this time-resolved transcriptomic study provides a descriptive analysis of the molecular responses of tobacco to dicamba stress. Our data suggest a putative three-stage adaptation pattern (“perception-defense-repair”), characterized by early activation of *NtIAA* genes and glutathione metabolism, mid-stage enrichment of photosynthetic and photoprotective pathways, and late-stage shifts toward secondary metabolism. Predictive regulatory network analysis screened the ERF transcription factor *Nitab4.5_0000015g0020* as a candidate hub gene hat may potentially link auxin signaling pathway and ribosomal protein gene expression. The *NtIAA* family is highlighted as a potential candidate of dicamba action, providing candidate genetic resources for further functional studies. This study offers a preliminary transcriptomic foundation for understanding dicamba phytotoxicity and for breeding herbicide-resistant crops.

## Data Availability

The datasets presented in this study can be found in online repositories. The names of the repository/repositories and accession number(s) can be found below: https://www.ncbi.nlm.nih.gov/genbank/, PRJNA1328999.

## Supplementary material

Supplementary Figure 1 Selection of WGCNA soft-thresholding power and scale−free topology validation. **(A)** Scale-free topology fit index (Signed R²) for different soft-thresholding powers (1-30). **(B)** Mean connectivity for different soft-thresholding powers. **(C)** Frequency distribution of connectivity (k) (log_10_ frequency vs. connectivity), showing fewer highly connected nodes, consistent with scale-free topology. **(D)** Linear fit of log_10_(p(k)) versus log_10_(k), confirming that the network at power = 30 exhibits good scale-free properties.

Supplementary Figure 2 GO and KEGG enrichment of co-expressed modules identified by WGCNA. **(A)** GO enrichment bubble plot for genes in the “greenyellow” module. **(B)** KEGG enrichment bubble plot for genes in the “greenyellow” module. **(C)** GO enrichment bubble plot for genes in the “navajowhite1” module. **(D)** GO enrichment bubble plot for genes in the “navajowhite1” module. **(E)** GO enrichment bubble plot for genes in the “salmon4” module. **(F)** GO enrichment bubble plot for genes in the “magenta4” module. The size of the dots indicates the number of genes enriched in the pathways. The color scale from blue to red indicates the significance of the enrichment results.

Supplementary Figure 3 The network structure of the co-expression modules that constructed using the top 50 genes based on intramodular connectivity. **(A)** Salmon4 module gene interaction network. Node color intensity reflects the magnitude of intramodular connectivity, with darker shades indicating higher topological importance. **(B)** Magenta4 module gene interaction network. **(C)** Navajowhite1 module gene interaction network. **(D)** Greenyellow module gene interaction network.
