# Supplementary figures and images for: Time-resolved transcriptomic analysis suggests candidate hub gene modules and putative regulatory pathways in tobacco under dicamba stress
Source: Front Plant Sci. 2026 Jul 13;17:1850240. doi: 10.3389/fpls.2026.1850240 (PMC13402193; doi:10.3389/fpls.2026.1850240)

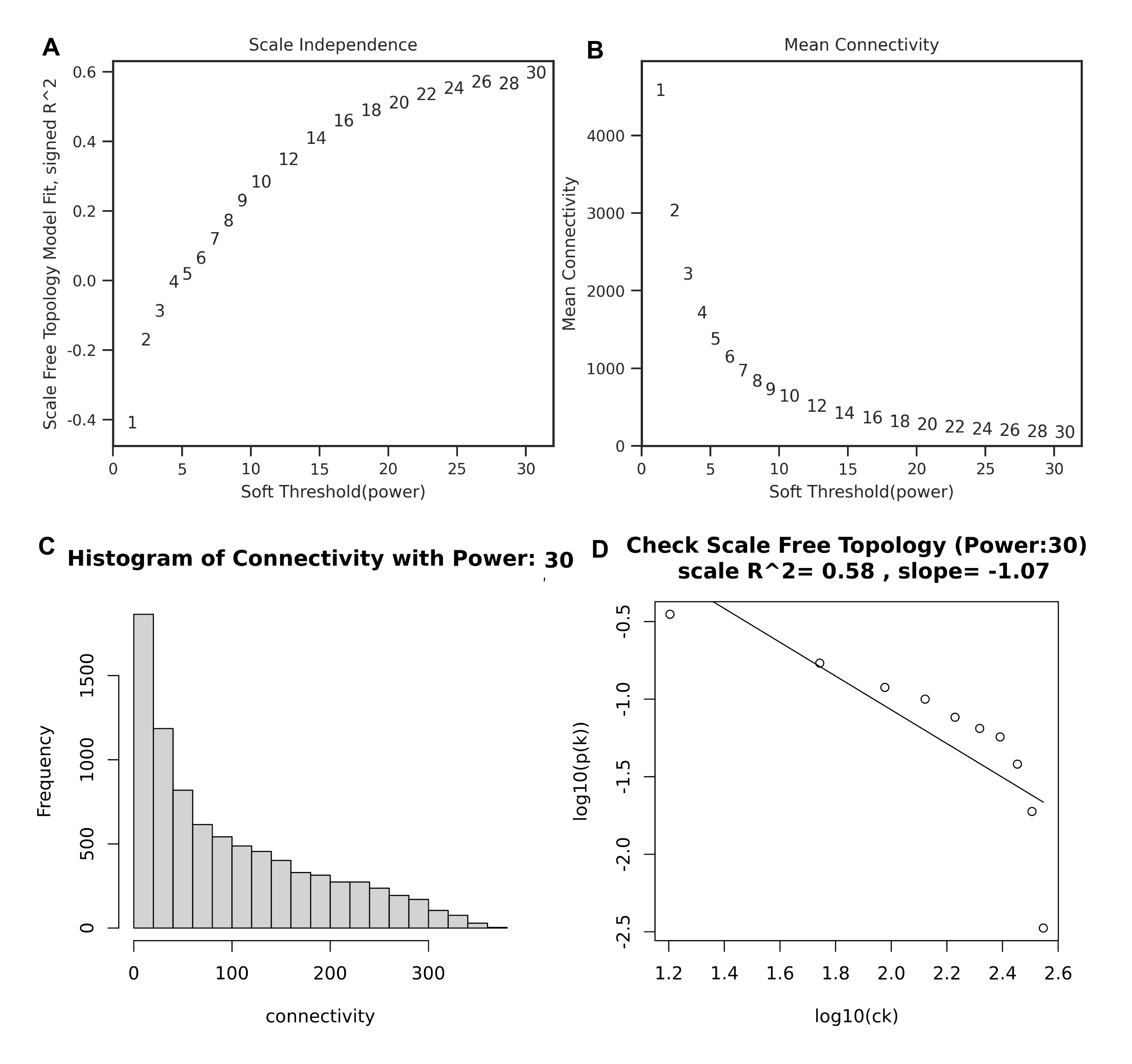

Supplement: Supplementary Figure 1 — Selection of WGCNA soft-thresholding power and scale−free topology validation. (A) Scale-free topology fit index (Signed R²) for different soft-thresholding powers (1-30). (B) Mean connectivity for different soft-thresholding powers. (C) Frequency distribution of connectivity (k) (log10 frequency vs. connectivity), showing fewer highly connected nodes, consistent with scale-free topology. (D) Linear fit of log10(p(k)) versus log10(k), confirming that the network at power = 30 exhibits good scale-free properties. [file Image1.jpeg]

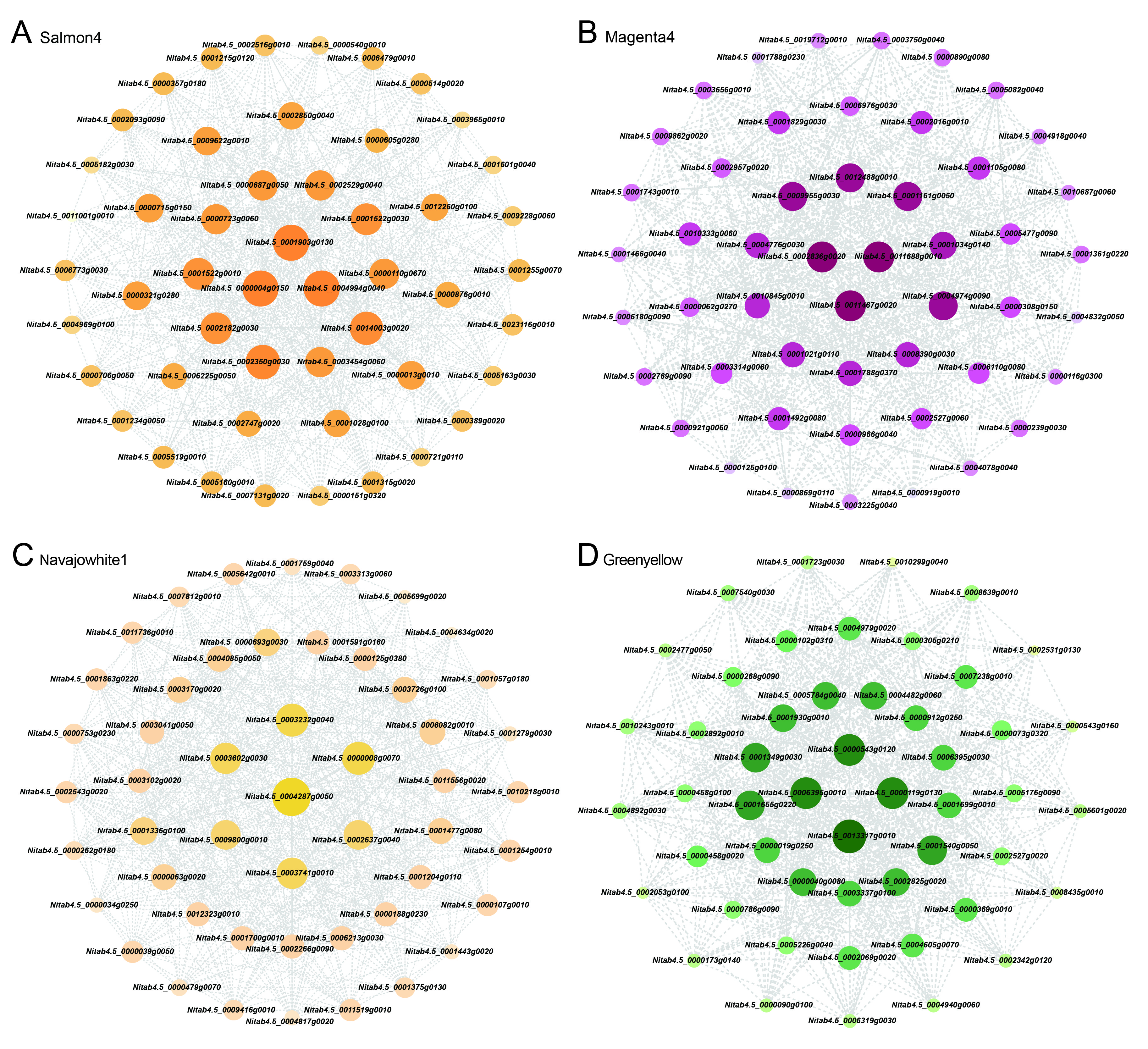

Supplement: Supplementary Figure 2 — GO and KEGG enrichment of co-expressed modules identified by WGCNA. (A) GO enrichment bubble plot for genes in the “greenyellow” module. (B) KEGG enrichment bubble plot for genes in the “greenyellow” module. (C) GO enrichment bubble plot for genes in the “navajowhite1” module. (D) GO enrichment bubble plot for genes in the “navajowhite1” module. (E) GO enrichment bubble plot for genes in the “salmon4” module. (F) GO enrichment bubble plot for genes in the “magenta4” module. The size of the dots indicates the number of genes enriched in the pathways. The color scale from blue to red indicates the significance of the enrichment results. [file Image2.jpeg]
